# The use of brewery waste as substrate on the lipid content and profile of the larvae *Tenebrio molitor*

**DOI:** 10.1007/s11250-026-05184-6

**Published:** 2026-06-22

**Authors:** Andressa Pelizari, Gabriel Nascimento de Souza Paulo, Rodrigo Borille, Caren Paludo Ghedini, Tatiana Emanuelli, Camila Sant’Anna Monteiro, Rafael Lazzari

**Affiliations:** 1 Departament of Animal Science, Center of Rural Sciences, Federal University of Santa Maria, Santa Maria, RS 97105-900 Brazil; 2 Faculty of Agricultural Sciences, Federal University of Grande Dourados, Dourados, MS 79825-900 Brazil; 3 Departament of Food Technology and Science, Center of Rural Sciences, Federal University of Santa Maria, Santa Maria, RS 97105-900 Brazil; 4 Department of Animal Science and Biological Sciences, Federal University of Santa Maria, Campus Palmeira das Missões, Palmeira das Missões, RS 98300-000 Brazil; 5 Faculty of Agronomy, Federal University of Rio Grande do Sul, UFRGS, Porto Alegre, RS 90046-900 Brazil; 6 Universidade Federal de Santa Maria, Avenida Roraima, 1000, Camobi, Santa Maria, 97105900 Brasil

**Keywords:** Insect fatty acids content, Alternative feedstuffs, Yellow mealworm, Animal nutrition

## Abstract

We investigated the effects of brewery waste as a substrate for rearing *Tenebrio molitor* on larvae growth performance and lipid composition. One thousand and five hundred *Tenebrio molitor* larvae were randomly assigned to five experimental treatments. Experimental treatments were created by replacing wheat bran with increasing levels of brewery waste: 0, 25, 50, 75 and 100%. *Tenebrio molitor* larvae growth performance was substantially changed by replacing wheat bran with incremental amounts of brewery waste, most notably by quadratic effects on larvae final body weight, daily body weight gain and substrate intake. Similarly, replacing wheat bran with incremental amounts of brewery waste linearly improved feed conversion ratio and reduced mortality of *Tenebrio molitor*. Larvae receiving 50% brewery waste had the highest final body weight, daily weight gain and substrate. Lipid content of *Tenebrio molitor* larvae increased linearly by replacing wheat bran with increasing amounts of brewery waste. Substantial changes were reported for larvae fatty acid profile. More notably, the use of brewery waste as an alternative rearing substrate increased stearic acid (C18:0) and palmitic acid (C16:0) content of *Tenebrio molitor* larvae. Myristic Acid (C14:0), Linoleic Acid (C18:2n6), Σ polyunsaturated and Σ n-3 polyunsaturated acid reduced linearly (*P* < 0.05) with adding incremental amounts of brewery waste. Whereas, pentadecanoic Acid (C15:0), Monounsaturated Heptadecanoic Acid (C17:1 n-5) and Linolenic Acid (C18:3 n-3) responded quadratically. Overall, our results indicate that brewery waste is a promising rearing substrate for *Tenebrio molitor* and markedly changes larvae lipid composition.

## Introduction

The growing demand for alternative protein sources, both for human and animal consumption, has encouraged research into the potential of using edible insects as a source of nutritious and sustainable food and feed. Among edible insects, *Tenebrio molitor*, also known as yellow mealworm, a beetle of the Tenebrionidae family, is one of the most promising insects to be used in animal production systems such as fish farming, due to its high nutritional value receiving great attention as a sustainable alternative feed to satisfy animal nutritional requirements improving their performance (Barroso et al. 2014; Veldkamp and Bosch 2015; Tubin et al. 2020).

Although mainly used as a source of high-quality protein, *Tenebrio molitor* is also a promising fat source for animal diets. Research has described *Tenebrio molitor* meal as a rich source of both, mono and polyunsaturated fatty acids (Pino Moreno and Ganguly 2016) which can be extremely beneficial in fish nutrition. Dietary lipids play a crucial role in providing essential fatty acids such as omega-3 and omega- 6 for the regulation of growth, health, reproduction, and bodily function in fish. Additionally, dietary fatty acids balance improves feed efficiency and overall nutritional quality of the diets. In this sense, *Tenebrio molitor* presents a great potential to be used as a lipid source in fish diets due to its high omega-3 and omega-6 FA content (Pino Moreno and Ganguly 2016).

Currently, fish oils are the lipid sources most commonly used in modern fish farming. These sources of fatty acids are rich in long-chain omega-3 fatty acids such as eicosapentaenoic acid (EPA) and docosahexaenoic acid (DHA). Although effective, the use of fish oil in fish diets faces challenges related to sustainability, such as overfishing and variable availability and price. Plant-based alternatives including flaxseed, canola and soybean oils are also used in fish nutrition, but they often contain short-chain omega-3 fatty acids such as alpha-linolenic acid (ALA), which needs to be endogenously converted to EPA and DHA, a process that can be inefficient. Therefore, lipid ingredients produced from insects are an emerging topic in animal nutrition, due to the numerous beneficial effects they can provide and *Tenebrio molitor* presents a promising source of fatty acids for fish nutrition (Errico et al. 2022), since several countries began to allow the use of insect meal in feed for commercial fish farming, following the approval of the European Commission in 2017 (Annex II of Regulation 2027/893 of May 24, 2017).

*Tenebrio molitor* larvae can grow on various substrates, including production waste, contributing to sustainability and waste reduction. Brewery waste, an abundant byproduct of the brewing industry is a rich source of fiber, protein and other nutrients and, therefore, presents a promising alternative rearing substrate for *Tenebrio molitor*. The use of waste as an alternative substrate in large-scale insect production not only promotes sustainability (Broekhoven et al. 2015), but can also influence the nutritional profile of larvae and thereafter, larvae meal, including its fat content and fatty acids profile (Anderson 2000; Broekhoven et al. 2015).

Despite the great potential of applying waste as substrate during edible insect rearing, studies investigating how brewery waste affects *Tenebrio molitor* larvae grow performance and lipid composition are still scarce in the current literature. Research in this regard are of great need to better understand the potential of waste-reared *Tenebrio molitor* larvae meal as feedstuff in animal production system including fish farming. Therefore, this study aimed to investigate the effects of using brewery waste as a substrate for rearing *Tenebrio molitor* on larvae growth performance and lipid composition.

## Materials and methods

This study was conducted at the Animal Nutrition Laboratory located at the Federal University of Santa Maria (UFSM), *campus* Palmeira das Missões, Rio Grande do Sul, Brazil. The first part of the study included all stages of *Tenebrio molitor* larvae development: reproduction, egg laying, and larvae growth up to 103 days (larvae production). Then, in the second part of the study increasing levels of brewery waste (experimental treatments) were used as a substrate for *Tenebrio molitor* larvae rearing for 28 days.

### Larvae production

To carry out mating and consequent laying of eggs on the substrate, 2000 adult beetles of *Tenebrio molitor* were kept into a clean plastic tray (57.0 × 37.0 × 14.0 cm) containing 500 g of wheat bran (substrate) during 7 days. On the last day of mating, all beetles were collected and the plastic tray with the remaining substrate and the eggs was then placed into an air-conditioned room (26.5 ± 1 °C and 65 ± 5% relative humidity) for 103 days after oviposition. On day 103, larvae averaging 40 mg were then selected (handpicked) to carry the second part of the study, as described below.

### Larvae rearing and experimental treatments

One thousand and five hundred *Tenebrio molitor* larvae with 103 days of age averaging 40 ± 2 mg/larvae (mean ± standard deviation) were selected (handpicked) from the growth tray and randomly assigned to 5 experimental treatments (300 larvae/treatment) with 5 replications each (60 larvae per sampling unit). Experimental treatments were created by replacing wheat bran (most commonly used substrate for *Tenebrio molitor* rearing) with increasing levels of brewery waste: 0, 25, 50, 75 and 100% (Table 1). Each sampling unit consisted of 60 larvae kept into clean plastic rearing containers, measuring 5.5 cm x 9.5 cm x 4.0 cm, containing 20 g of substrate (experimental treatments). Substrate was provided *ad libitum* in all periods for all sampling units. The evaluation of *Tenebrio molitor* larvae grow performance was carried over a period of 28 days in which by the end of the performance evaluation larvae age was 131 days.

**Table 1:** Experimental treatments (rearing substrate) composition

Ingredients	Experimental treatments				
	0%	25%	50%	75%	100%
Wheat bran (%)	100	75	50	25	0
Brewery waste (%)	0	25	50	75	100
	**Nutritional composition**				
Gross energy (Mcal/kg)	39.22	41.39	43.57	45.75	47.92
Crude fiber (%)	9.00	10.26	11.51	12.77	14.03
Ether Extract (%)	3.40	4,54	5.68	6,82	7.96
Crude protein (%)	15.10	17.18	19.27	21.35	23.44

### Larvae growth performance evaluation

The following response variables were collected to evaluate larvae growth performance: initial live body weight (mg/larvae), final live body weight (mg/larvae), daily weight gain (mg/larvae/day), substrate intake (mg/larvae/day), feed conversion ratio (mg/mg), feces production (mg/larvae/day) and larvae mortality (%) at day 28. To obtain the initial and final live weight, the larvae were weighed at the beginning (day 1) and at end (day 28) of the experimental period and, subsequently, the value was divided by the number of larvae in each rearing container. Daily live weight gain per larva per day was obtained by subtracting the initial live weight from the final live weight and dividing the value by the number of days of the experimental period (28 days).

To measure daily substrate intake, 20 g of substrate was added to each rearing container at the begging of the study and the remaining substrate (substrate orts) were collected weekly and adjusted to the number of live larvae. At the end of the experimental period (day 28), weekly intake was then added to obtain the accumulated intake (28 days intake). Then, accumulated intake of substrate was divided by 28 to obtain daily substrate intake. Feed conversion ratio was established by the ratio between substrate intake (mg/larvae/day) and weight gain (mg/larvae/day). Larvae mortality percentage was obtained by multiplying the number of dead larvae by 100 and dividing the result by the initial number of live larvae allocated into each rearing container (300 larvae). All larvae grow performance measurements were performed using a 0.001 g precision balance (model BELMG214Ai, 0.1 mg precision).

### Collection and preparation of samples for laboratory analysis

At the end of the experimental period (131 days after oviposition), larvae were separated from the substrate, counted and weighed (final live weight) in their sampling units. Then, larvae were placed back to the plastic rearing containers for 24 h for fasting, with the aim of cleaning their digestive tract. After fasting, larvae were collected and packaged in plastic bags, and then placed in a freezer for euthanasia by freezing. Three days after freezing, the larvae were sent for identification and quantification of fatty acids.

### Total lipids and fatty acid profile

The total lipid content of samples of *Tenebrio molitor* larvae was extracted using chloroform and methanol, cold, using the Bligh-Dyer method (1959), and quantified by gravimetric analysis. To prevent lipid oxidation of samples during and after extraction, 0.02% Butylhydroxytoluene (BHT) was used together with chloroform. The chloroform phase was also used to analyze the composition of fatty acids after being saponified in a methanolic KOH solution and esterified in a methanolic H2SO4 solution (Hartmann & Lago 1973).

Methylated fatty acids were analyzed using an Agilent Technologies gas chromatograph (HP 6890 N) equipped with a capillary column (DB-23 60 m x 0.25 mm x 0.25 μm) and flame ionization detector. The injector port temperature was set at 250 °C and the carrier gas was nitrogen (0.6 mL/min). After injection (1 µL, split ratio 50:1), the oven temperature was maintained at 150 °C for 1 min, was increased to 240 °C at 4 °C/min, and held at that temperature for 12 min. Standard fatty acid methyl esters (37-component FAME Mix from Sigma, Saint Louis, MO, USA) were run under the same conditions and subsequent retention times were used to identify the fatty acids. Fatty acids were expressed as a percentage of the total fatty acids identified.

### Experimental design and statistical analyzes

The experiment was carried out in a completely randomized design, with 5 treatments and 5 replications of 60 larvae each, totaling 25 experimental units. Data were subjected to normality tests and subsequently to ANOVA. Polynomial regression models were applied, with the levels of replacement of wheat bran by brewery waste considered as independent variables. The equations that showed significant effects (*P* < 0.05) were selected. All statistical analyzes were performed using Minitab 17 statistical software.

## Results

### Effects on larvae growth performance

*Tenebrio molitor* larvae growth performance was substantially changed by replacing wheat bran with incremental amounts of brewery waste, most notably by quadratic effects on larvae final body weight, daily body weight gain and daily substrate intake. Similarly, replacing wheat bran with incremental amounts of brewery waste linearly reduced feed conversion ratio and mortality of *Tenebrio molitor* larvae (Table 2). Regarding brewery waste levels, it was observed that *Tenebrio molitor* larvae receiving 50% brewery waste had the highest final live body weight (77.53 mg/larvae), daily weight gain (1.05 mg/larvae/day) and substrate intake (5.84 mg/larvae/day).

**Table 2:** Effects of replacing wheat bran with increasing levels of brewery waste on *Tenebrio molito*r larvae growth performance

Variable	0%	25%	50%	75%	100%	SEM	*P*-Valor
Initial live body weight (mg/larvae)	45,78	47,12	47,98	44,187	45,67	0,972	0,099
Final live body weight (mg/larvae)	67,41	73,48	77,53	70,40	70,17	1,560	0,002
Body weight gain (mg/larvae/day)	0,77	0,94	1,05	0,93	0,87	0,032	< 0,001
Substrate intake (mg/larvae/day)	5,18	5,56	5,84	5,47	4,91	0,145	0,002
Feces production (mg/larvae/day)	6,22	7,36	8,75	10,55	9,64	0,394	< 0,001
Food conversion ratio (mg/mg)	6,80	5,92	5,55	5,87	5,64	0,285	0,041
Larvae Mortality (%, at 28 days)	0,34	0,00	0,00	6,60	3,95	1,210	0,002
	**Model**				**r**	**R2**	**P-Valor**
Final live body weight (mg/larvae)	Y = 67,91 + 0,2817 X − 0,002720 X²				-	0,378	0,005
Body weight gain (mg/larvae/day)	Y = 0,7769 + 0,008730 X − 0,000079 X²				-	0,601	< 0,001
Substrate intake (mg/larvae/day)	Y = 5,162 + 0,02644 X − 0,000290 X²				-	0,535	< 0,001
Feces production (mg/larvae/day)	Y = 5,975 + 0,08245 X − 0,000424 X²				-	0,726	< 0,001
Food conversion ratio (mg/mg)	Y = 6,436–0,009529 X				-0,47	0,217	0,019
Larvae mortality (%, at 28 days)	Y = − 0,584 + 0,05528 X				+ 0,54	0,293	0,005

Feces production increased significantly when brewery waste replaced wheat bran, notably at levels of 75% and 100% (Y = 5.975 + 0.08245x − 0.000424x²; *P* < 0.001; R² = 0.7267). A decreasing linear behavior was observed for feed conversion ratio when increasing levels of brewery waste were used as an alternative substrate for *Tenebrio molitor* rearing (Y = 6.436–0.009529X; *P* < 0.05; R² = 0.2178; *r* = -0.47), showing that the level of replacement can provide a relative tendency to improve substrate bioconversion by *Tenebrio molitor* larvae, as despite being significant, the increase in the levels of replacement of wheat bran by brewery waste had little influence (R² = 0.21) on feed conversion ratio. Larvae mortality was significantly higher with 75% and 100% brewery waste compared to other levels of replacement.

### Lipid profile

Lipid content of *Tenebrio molitor* larvae meal increased linearly by replacing wheat bran with increasing amounts of brewery waste (Table 3). Similarly, Palmitic Acid (C16:0) and Stearic Acid (C18:0) concentration in *Tenebrio molitor* larvae increased linearly with incremental amounts of brewery waste. Conversely, no changes were reported for Lauric Acid (C12:0), Heptadecanoic Acid (C17:0), Arachidic Acid (C20:0), Monounsaturated Palmitic Acid (C16:1 n-7), Oleic Acid (C18:1 n-9), Eicosenoic Acid (C20:1 n-9), Eicosadienoic Acid (C20:2 n-6), Σ saturated and Σ Monounsaturated with replacing wheat bran by brewery waste (Table 3). On the other hand, Myristic Acid (C14:0), Linoleic Acid (C18:2n6), Σ polyunsaturated and Σ n-3 polyunsaturated acid reduced linearly (*P* < 0.05) with adding incremental amounts of brewery waste. Pentadecanoic Acid (C15:0), Monounsaturated Heptadecanoic Acid (C17:1 n-5) and Linolenic Acid (C18:3 n-3) responded quadratically when feeding *Tenebrio molitor* larvae with incremental amounts of brewery waste as an alternative rearing substrate.

**Table 3:** Effects of replacing wheat bran with increasing levels of brewery waste on *Tenebrio molitor* larvae lipid content and fatty acid composition

Variable	Experimental treatments						
	0	25	50	75	100	CV	*P*-valor
Total lipids, g/100 g	4,4	4,25	4,6	4,6	4,9	2,5	0,01
Fatty acids, g/100 g							
C12:0	0,34	0,33	0,32	0,28	0,26	11,33	0,257
C14:0	5,32	5,45	4,96	4,60	4,57	2,79	0,004
C15:0	0,1	0,09	0,08	0,08	0,08	3,63	0,004
C16:0	15,72	16,4	17	17,2	17,7	1,84	0,009
C17:0	0,1	0,09	0,08	0,1	0,12	20,4	0,582
C18:0	2,9	3,1	3,25	3,48	3,38	3,38	0,016
C20:0	0,08	0,17	0,19	0,19	0,2	33,83	0,338
∑ Saturated	24,56	25,7	25,8	25,9	26,3	1,82	0,082
C16:1n-7	2,335	2,32	2,28	2,12	2,31	5,27	0,47
C17:1n-5	0,12	0,11	0,11	0,11	0,12	3,85	0,038
C18:1n-9	47,18	46,7	47,5	47,1	46,8	1,23	0,663
C20:1n-9	0,09	0,09	0,09	0,09	0,1	13,16	0,892
∑ Mono	49,73	49,2	50	49,4	49,3	1,07	0,602
C18:2n-6	25,04	24,5	23,6	24,1	23,7	0,87	0,005
C18:3n-3	0,64	0,56	0,53	0,55	0,61	3,49	0,021
C20:2n-6	0,03	0,06	0,05	0,04	0,05	75,69	0,968
∑ PUFA	25,71	25,1	24,2	24,7	24,4	0,95	0,007
∑ n-3	0,64	0,56	0,53	0,56	0,61	3,49	0,021
∑ n-6	25,07	24,6	23,7	24,2	23,8	0,94	0,007
**Variable**	**Mathematical model**				**r**	**R²**	**P valor**
Total lipids, g/100 mg	Y = 4,307 + 0,005093 X				+ 0,79	0,62	0,006
C14:0	Y = 5,450–0,009421 X				-0,89	0,79	< 0,001
C15:0	Y = 0,1031–0,000580 X + 0,000004 X²				-	0,91	< 0,001
C16:0	Y = 15,85 + 0,01876 X				+ 0,94	0,87	< 0,001
C17:1n5	Y = 0,1225–0,000424 X + 0,000004 X²				-	0,79	0,004
C18:0	Y = 2,952 + 0,005439 X				+ 0,87	0,75	0,001
C18:2n6	Y = 24,81 − 0,01213 X				-0,79	0,60	0,008
C18:3n3	Y = 0,6382–0,003868 X + 0,000036 X²					0,85	0,001
Σ Polyunsaturated	Y = 25,45 − 0,01237 X				-0,76	0,57	0,01
Σ Polyunsaturated n-3	Y = 0,6382–0,003868 X + 0,000036 X²				-	0,85	0,001
Σ Polyunsaturated n-6	Y = 24,85 − 0,01209 X				-0,78	0,60	0,008

The greatest contents of C15:0, C17:1n-5 and C18:3n-3 were reported in *Tenebrio molitor* larvae fed no brewery bran (0% treatment), whereas the lowest levels of these fatty acids were observed with including 75%, 50% and 50% brewery waste to the rearing containers, respectively.

## Discussion

The standard diet for *Tenebrio molitor* is based on wheat bran. However, using wheat bran as a substrate for insect rearing can be unaffordable especially in developing countries due to its expensive cost. In this sense, there has been a continuous interest in seeking for alternative rearing substrate for insect production including *Tenebrio molitor*. Recent research have indicated that wheat bran promotes a greater grow performance of *Tenebrio molitor* than alternative rearing substrates (El Deen and Lamaj 2021; Langston et al. 2024). For instance, according to Langston et al. (2024) wheat bran fed *Tenebrio molitor* were five times heavier than those receiving wheat flour, maize flour, lucerne and oats as alternative substrate, indicating the effectiveness of wheat bran a substrate for rearing *Tenebrio molitor*.

The ability of insects in converting low quality substrates increases their potential to be used as an alternative sustainable ingredient in human and animal diets. Indeed, our results indicate that brewery waste can be used as an alternative rearing substrate for *Tenebrio molitor* as larvae growth performance was notably improved with replacing wheat bran by brewery waste (Table 2). Nutritional composition of the substrate increased expressively with adding increasing amounts of brewery waste. Substrate energy, fiber, fat and protein content increased with adding incremental amounts of brewery bran into the rearing containers (Table 1). Therefore, improvements on larvae growth performance are likely linked to increased substrate nutritional composition provided during larvae rearing. According to our results, wheat bran can be replaced with brewery waste up to 50%. The increase in larval growth performance variables up to 50% replacement of the standard diet suggests that partial replacement of wheat bran with brewery waste can optimize larval growth, possibly by providing a better nutritional balance when offered in this proportion (50% brewery waste plus 50% wheat bran).

According to Morales-Ramos et al. (2011) and Fasce et al. (2022) wheat bran is an effective substrate for *Tenebrio molitor* earing since it adequately adapts to *Tenebrio molitor* feeding behavior and is generally self-selected by this insect to meet nutritional needs. However, Fasce et al. (2022) also suggests that feeding *Tenebrio molitor* with a single feed, such as wheat bran, increases the risk of nutritional imbalances that may limit its growth performance. These authors further indicated that supplementing wheat bran with other concentrate substrates improves *Tenebrio molitor* performance by reducing fiber and increasing essential nutrients offered to the larvae. Similarly, the study conducted by El Deen et al. (2021) showed that rearing *Tenebrio molitor* on wheat bran supplemented with yeast improved adult survival and increased number of larvae probably by providing a more balanced diet than only wheat bran. These findings indicate the effectiveness of supplementing alternative substrate sources (i.e. brewery waste and yeast) together with standard wheat bran for *Tenebrio molitor* performance. Therefore, improvements in daily weight gain, live final weight and substrate intake reported herein with replacing wheat bran by brewery waste at 50% replacement may be related to both, the quantity and quality of nutrients in the diet offered to *Tenebrio molitor*, such as protein and starch (Morales-Ramos et al. 2010; Van Broekhoven et al. 2015; Fasce et al. 2022) which, consequently, may favor the absorption of essential nutrients or, even, could had complemented larvae nutritional needs more efficiently than isolated wheat bran, since brewery waste is a product originating from a cereal that has undergone a fermentation process which changes its chemical structures. Our results are similar to those reported by Broekhoven et al. (2015) where replacing wheat bran with 40% brewery waste reduced growth time of the larvae until the pupa stage and increased pupae weight of *Tenebrio molitor*.

In our study, larvae receiving 50% brewery waste had the highest substrate intake indicating that possibly nutrient balance offered to the *Tenebrio molitor* colony was greater when rearing substrate was composed by 50% wheat bran and 50%brewery waste. In this sense, the likely improved nutrient balance at this substrate (wheat bran and brewery waste) combination may had encouraged a greater intake of substrate, increasing larvae daily weight gain by providing greater amounts of energy and protein to the larvae and finally allowing greater final live weight. Although partial replacement improves performance, very high levels of brewery waste (above 50%) can lead to reduced substrate use by the larvae which, consequently, generates greater excretion of undigested material. Substrate fiber content increased as brewery waste replaced wheat bran at increasing amounts which may have negatively impacted substrate digestibility. Indeed, we reported that feces production increased with including brewery waste (Tables 1 and 2). In this context, Fasce et al. (2022) carried out a digestibility test in a study carried to evaluate the inclusion of carrots and wet brewery waste in diets of *Tenebrio molitor* larvae and mentioned that the high dietary fiber (NDF and lignin) may be negatively related to digestibility of larvae. This suggests an increase in feces production, supporting the results observed in this study, however, it is important to highlight that no digestibility analysis was performed herein to prove this fact. Bioconversion of *Tenebrio molitor* increased when brewery waste was used as a rearing substrate as indicated by the results in food conversion ratio (Table 2). Similarly, Fasce et al. (2022) observed that the addition of wet brewery waste improved food conversion ratio of *Tenebrio monitor* larvae in relation to providing only wheat bran as a rearing substrate.

In addition to ensure larvae growth performance, our results showed that using brewery waste as a rearing substrate for *Tenebrio molitor* improves its potential to be used as an alternative lipid source in animal production systems, such as fish farming as larvae lipid content increased linearly with including incremental amounts or brewery waste. Substrate fat content increased as incremental amounts of brewery waste replaced wheat bran (Table 1) explaining the greater larvae lipid content and indicating the potential of *Tenebrio molitor* of using substrate lipid sources as lipid deposition increased (greater fat content, Table 3) when incremental amounts of lipids were offered to the larvae. Furthermore, are results indicated that fatty acid profile of *Tenebrio molitor* larvae substantially changes with replacing wheat bran by brewery waste. Notably the use of brewery waste as a rearing substrate increases stearic acid (C18:0) and palmitic acid (C16:0) content of *Tenebrio molitor* larvae. Similar to our results, the study conducted by Boukid et al. (2021) evaluating different agro-industrial by-products as alternative substrate for rearing different insect species, including *Tenebrio molitor sp.* reported markedly changes in insect fatty acid depending on rearing substrate composition. These finding indicate that the fatty acid profile of *Tenebrio molitor* is significantly influenced by substrate composition, consistent with previous studies carried out with different substrates, including chia seeds, flax seeds, oat or wheat flours or vegetables (Ravzanaadii et al. 2012; Dreassi et al. 2017; Lawal et al. 2021).

Overall, our results suggest that brewery waste is a promising sustainable substrate for *Tenebrio molitor* rearing and offering 50% wheat bran plus 50% brewery waste as a substrate enhances bioconversion of substrate by *Tenebrio Molitor* larvae. Furthermore, replacing standard wheat bran with brewery waste changes fatty acid composition of *Tenebrio molitor* larvae. Future studies should continue to explore the interactions between different rearing substrates and their impacts on *Tenebrio molitor* larval physiology and productivity as the ability of insects in converting low quality products, such as waste, into high quality feed or food is remarkedly linked to sustainability of insects as an alternative source in both, animal and human diets.

## Data Availability

Data available on request from the authors.
